# The Role of Exosomal microRNAs and Oxidative Stress in Neurodegenerative Diseases

**DOI:** 10.1155/2020/3232869

**Published:** 2020-10-17

**Authors:** Xiaoyu Wang, Yunxiang Zhou, Qiannan Gao, Dongnan Ping, Yali Wang, Wei Wu, Xu Lin, Yuanjian Fang, Jianmin Zhang, Anwen Shao

**Affiliations:** ^1^Department of Neurosurgery, The Second Affiliated Hospital, School of Medicine, Zhejiang University, Hangzhou 310009, China; ^2^Department of Surgical Oncology, The Second Affiliated Hospital, School of Medicine, Zhejiang University, Hangzhou, Zhejiang 310009, China; ^3^State Key Laboratory of Cardiovascular Disease, Fuwai Hospital, National Center for Cardiovascular Diseases, Chinese Academy of Medical Sciences and Peking Union Medical College, Beijing 100037, China; ^4^Sir Run Run Shaw Hospital, Zhejiang University School of Medicine, Hangzhou, Zhejiang 310016, China; ^5^The First Affiliated Hospital, College of Medicine, Zhejiang University, Hangzhou 310003, China

## Abstract

Neurodegenerative diseases including Alzheimer's disease and Parkinson's disease are aging-associated diseases with irreversible damage of brain tissue. Oxidative stress is commonly detected in neurodegenerative diseases and related to neuronal injury and pathological progress. Exosome, one of the extracellular vesicles, is demonstrated to carry microRNAs (miRNAs) and build up a cell-cell communication in neurons. Recent research has found that exosomal miRNAs regulate the activity of multiple physiological pathways, including the oxidative stress response, in neurodegenerative diseases. Here, we review the role of exosomal miRNAs and oxidative stress in neurodegenerative diseases. Firstly, we explore the relationship between oxidative stress and neurodegenerative diseases. Secondly, we introduce the characteristics of exosomes and roles of exosome-related miRNAs. Thirdly, we summarized the crosstalk between exosomal miRNAs and oxidative stress in neurodegenerative diseases. Fourthly, we discuss the potential of exosomes to be a biomarker in neurodegenerative diseases. Finally, we summarize the advantages of exosome-based delivery and present situation of research on exosome-based delivery of therapeutic miRNA. Our work is aimed at probing and reinforcing the recognition of the pathomechanism of neurodegenerative diseases and providing the basis for novel strategies of clinical diagnosis and treatment.

## 1. Introduction

The incidence of neurodegenerative diseases, which include Alzheimer's disease (AD), Parkinson's disease (PD), Huntington's disease (HD), and amyotrophic lateral sclerosis (ALS), has been shooting up due to the extended lifespan and environment pollution. Neurodegenerative diseases are a group of refractory diseases and have loaded a huge medical, social, and economic burden to the world. Despite the massive efforts into the pathological mechanisms and therapeutic strategies of neurodegenerative diseases, few sufficiently effective treatments have been generated thus far [1]. Most neurodegenerative diseases are inherited diseases with some genome mutant in the neurons; meanwhile, environmental insults are also fundamental to the disease progression. Accumulating evidence has shown that the pathology of neurodegenerative diseases has a strong contact with the production of oxidative stress, which in turn, contributes to the further progress of neurodegenerative diseases [2–4]. Oxidative stress is characterized as the imbalance between the production of reactive oxygen species (ROS) and the ability to scavenge them [5, 6]. The ROS accumulation in neurons can induce mitochondria dysfunction and cell apoptosis, thereby yielding neuronal injury [7].

Exosomes are extracellular vesicles (EVs) secreted by a variety of cells and carry cargos including protein, lipid, and noncoding RNA (ncRNA) [e.g., long noncoding RNA (lncRNA), microRNA (miRNA), and messenger RNA (mRNA)]. Following the release, exosomes will transfer to specific targets such as immune cells or the central nervous system (CNS) to exert pleiotropic effects [8, 9]. In this context, exosomes can take part in many biological processes and set up intracellular communication among cells, which makes them important in diverse diseases, e.g., immunological diseases, tumorigenesis, and neurodegenerative diseases [10]. Exosomes involved in neurodegenerative diseases are generated from manifold sources, such as human mesenchymal stem cells (MSCs), immunity cells, and microglia [9, 11–14], and exosomes derived from different sources with different cargos seem to have a different impact on neurodegenerative diseases [15]. Notably, exosome-derived miRNAs have the potential to interact with oxidative stress response during the neurodegenerative processes [16]. More importantly, exogenous exosomes can cross the blood-brain barrier (BBB) and target the brain tissue [17], while endogenous exosomes can be secreted by brain cells and reflect brain injury [9], indicating their promise as drug carriers and biomarkers for neurodegenerative diseases, respectively. Accordingly, this review attempts to briefly summarize the potential advantage of exosomal miRNA-based management in the treatment and diagnosis of neurodegenerative diseases.

## 2. Oxidative Stress and Neurodegenerative Diseases

Oxidative stress is a reactive process posed by the aggregation of free radicals arising from the changed environment including inflammation and mitochondria dysfunction [18]. Reactive oxygen species (ROS) are identified as fundamental free radicals exacerbating oxidative stress and aggravating tissue dysfunction [19]. The production and clearance process of ROS are a dynamic balance *in vivo*. In a normal situation, the proper ROS level is thought to be necessary to maintain the activation of certain signaling pathways (e.g., EGFR pathway, Ras/AMPK pathway, and PKC pathway), stimulate the cell proliferation, and regulate the cell metabolism. However, when the production of ROS seriously exceeds the scavenging capacity, the ROS will accumulate and have an effect on cells, leading to DNA [nuclear and mitochondria DNA (mtDNA)] damage, protein misfolding, and chromosome instability, among others [20–23].

**Figure 1:**
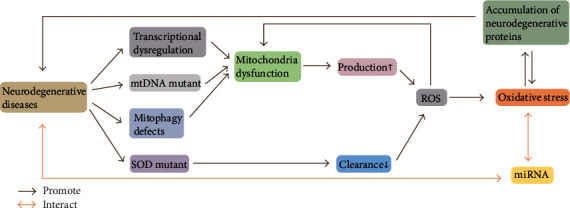
Oxidative stress and miRNAs are critically involved in the pathogenesis of neurodegenerative diseases. The pathology of neurodegenerative diseases is closely related to the generation of oxidative stress, which in turn promotes the further progression of neurodegenerative diseases. miRNAs can interact with the oxidative stress response and other pathophysiological processes underlying neurodegenerative diseases.

Neurodegenerative diseases are specifically characterized by apoptosis/necrosis and dysfunction of neuronal cells, leading to compromised motor or cognitive function. Given its high metabolic rate and high-lipid content, CNS is particularly vulnerable to oxidative stress, and the relationship between the neurodegenerative diseases and oxidative stress, therefore, has attracted great interest. Correspondingly, accumulating evidence has shown that oxidative stress is critically involved in the pathogenesis of neurodegenerative diseases as high levels of oxidative stress are commonly observed in the brain of patients with neurodegenerative conditions [24, 25] and may represent one of the potential pathological processes for targeted intervention (Figure 1) [26, 27]. In this section, we focus on the critical role of ROS and oxidative damage in major neurodegenerative diseases including AD, PD, HD, and ALS and discuss in-depth the latest and most recent advances in the field of neurodegenerative diseases.

### 2.1. Alzheimer's Disease (AD)

AD is characterized by the pathological accumulation of A*β* and resultant cerebral amyloid angiopathy, neurofibrillary tangles comprising hyperphosphorylated neuronal tau, and neuronal loss [28]. The production of A*β* peptide and oxidative stress seems to be inseparable. Although the determinant of A*β* production is aging, studies have shown that A*β* can be induced when the brain is exposed in an environment of ROS overload, further bringing about the development of AD [29, 30]. The mechanisms through which oxidative stress triggers the A*β* production remain elusive, and some research showed that the oxidative stress contributed to cerebral A*β* production and accumulation in A*β*-rich environment through the p38 mitogen-activated protein kinase signaling pathway [31], the nuclear factor-*κ*-gene binding pathway activation [32], or the increase of lipid peroxidation [33]. Reciprocally, A*β* has several pathways to induce cells to overexpress ROS and then increase oxidative stress. For instance, metal ion-chelate A*β* can restore the O_2_ through a three-step cycle where O_2_ is gradually reduced to superoxide and oxygen peroxide, eventually forming OH radicals and generating ROS as byproducts [34–36]. Moreover, A*β* can also directly stimulate oxidative stress through endoplasmic stress, lipid peroxidation, and mitochondria dysfunction [37–39]. Furthermore, researchers suggest that it is the monomers and small oligomer A*β*, rather than A*β* plaques, that induce oxidative stress and result in the cell toxicity and neuron injury [40].

### 2.2. Parkinson's Disease (PD)

Compared to AD, PD is more relevant to oxidative stress. PD is a multifactorial neurodegenerative disease with the impairment of voluntary motor control evolving over time and has a preferential dopaminergic neuronal loss in the substantia nigra. PD patients present with a wide range of motor symptoms including postural instability, bradykinesia, tremor, and rigidity. Histopathologically, *α*-synuclein boosts the formation of the Lewy bodies and Lewy neurites in the brain, which are also a hallmark of PD and can be induced by oxidative stress [41–43]. Although the exact etiology and natural course of this disease have yet been fully determined, it appears likely that dysfunction of numerous processes, such as mitochondria functioning, autophagy, dopamine homeostasis, and calcium homeostasis, is strictly involved [44]. In the pathogenesis of PD, the mitochondria dysfunction is the major source of ROS, and mitochondria are, in turn, the targets of ROS [45].

mtDNA mutant is the first step of mitochondria dysfunction. The mtDNA is partly independent of nuclear DNA. Although the nuclear DNA encodes most of the proteins needed for mitochondria functioning, the mtDNA itself also encodes the essential protein and RNAs, such as cyclooxygenase (COX) and ribosome, in the mitochondrial respiratory chain. Thus, mtDNA is indispensable in the mitochondria function. In the patient with PD, the mtDNA in neurons, especially in nigra, is found to have a high level of mutant and deletion, which may explain the involvement of oxidative stress in PD [46, 47]. In addition, mitophagy defects are present in PD patients. In this case, mitophagy cannot process correctly due to the mutant mitophagy-relevant genes, leading to the accumulation of impaired mitochondria and eventually inducing the pathological process of PD. These mutant genes include *LRRK2*, *PINK1*, *Parkin*, and *DJ-1*, among which *PINK1/Parkin* mutant primarily contributes to the mitophagy deficiency and ensuing oxidative stress [48, 49].

### 2.3. Huntington's Disease (HD)

HD is a human autosomal dominant neurodegenerative disease with a CAG repeat expansion mutation in the exon 1 of the *Huntingtin* gene (*Htt*) [50]. In the clinic, patients with HD have some characteristic behaviors including choreiform movements, behavioral abnormalities, and cognitive decline. Some research has shown that the mutant Huntingtin protein (mHtt) has proteotoxicity and is the major cause of HD development. The expression of mHtt is influenced by some ecological factors such as age and environment, mainly age [51]. Importantly, mHtt can induce multiple injurious effects, including aberrant gene transcription, defective autophagy, abnormal mitochondrial biogenesis, and anomalous mitochondrial dynamics and trafficking, which will impair the oxidative metabolism, generate ROS, and finally cause the neuron damage and death [52]. The correlation between mHtt and oxidative stress is principally reflected in mitochondria impairment and Ca^2+^ handling. Studies have found that mHtt could alter the mitochondria dynamic (fusion and fission) and further damage the mitochondria morphology through oxidative stress, leading to the apparent mitochondrial fragmentation and dysfunction [53–55]. Moreover, some researchers argued that mHtt indirectly impairs mitochondrial function by hindering the mitophagy, as the induced mitochondria dysfunction can be relieved by the overexpression of PINK1, which regulates Parkin-mediated mitophagy [49]. In terms of Ca^2+^ handling, mHtt can increase the intracellular Ca^2+^ loading and cause a transcriptional dysregulation, resulting in the mitochondrial impairment which includes a decrease in mitochondrial Ca^2+^ uptake capacity, ATP production, and ROS defense [56].

### 2.4. Amyotrophic Lateral Sclerosis (ALS)

ALS, also named motor neuron disease, Lou Gehrig disease, or Charcot disease, is a kind of serious devastating neurodegenerative disease. ALS mainly involves the motor neurons regardless of the upper or lower ones. The progressive motor deficits are the characteristic symptom of patients with ALS, quickly spreading from focal to other body regions in weeks or months. Patients usually die from the complication and paralysis of skeletal muscles, particularly bulbar and respiratory muscles [57]. Quite a lot of studies have indicated that oxidative stress is critically implicated in ALS, given that oxidative stress has already been recognized as a biomarker of ALS and oxidative stress seems to increase the neuronal death and boost the ALS progress [58]. However, how oxidative stress is triggered and subsequently accelerates the ALS pathogenesis is still far away from understanding. Indeed, approximately 10% of ALS cases are familial and 20% of those cases have a mutant of Cu/Zn superoxide dismutase (SOD1), a member of the SOD family, which are the major enzymes to scavenge ROS. Thus, the mutant of SOD1 is adequate to induce oxidative stress and damage the neurons. Correspondingly, in animal models of ALS, the SOD1 mutant exactly changes the CNS lipid peroxidation, increases the ROS, and damages the cells irrespective of neurons or muscular cells [59]. Furthermore, antioxidant drugs or restoring the SOD1 can reverse these processes, relieve the ALS progress, and extend the ALS model lifespan [60, 61]. Similar to the other neurodegenerative diseases, mitochondria dysfunction is also a probable source of oxidative stress in ALS [62]. Some studies suggest that lifestyle changes and more exercise can have beneficial effects on ALS by ameliorating oxidative stress [63, 64]. Taken collectively, the oxidative stress response can be a candidate for precaution, biomarker, and therapeutic target of ALS.

## 3. Exosomes and Exosome-Associated miRNA

### 3.1. The Definition, Generation, Transport, and Biology of Exosomes

**Figure 2:**
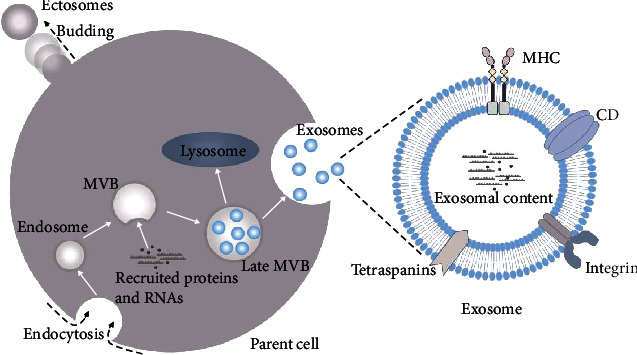
Schematic representation of the formation and composition of extracellular vesicles. Ectosomes are generated by direct outward budding of the plasma membrane, while exosomes are derived from endosomes. The plasma membrane of the parent cell forms the endocytic vesicles (endosomes), which inward bud and recruit protein and RNA cargo to form multivesicular bodies (MVBs). Eventually, MVBs fuse with the plasma membrane to release cargo-enriched exosomes into the extracellular space or get degraded by lysosomes. The membrane-type structure of the exosome is made of a lipid bilayer. Exosomes encompass cytosol of the parent cell from which they are derived and express the extracellular domain of distinct transmembrane proteins, such as integrin, tetraspanins, major histocompatibility complex (MHC), and cluster of differentiation (CD), which reflect the type of parent cell.

The EVs are some kinds of vesicles actively released by a variety of mammal cells. These vesicles are heterogeneous and made of membrane-bound phospholipids and have numerous functions [65]. The EVs are divided into two major species, the ectosomes and exosomes, although sometimes they are thought to be the same kind of vesicle. Indeed, they are quite different in location, size, markers, and other aspects [66]. Since first introduced in 1970 [67], exosomes have been studied for quite a long time. Exosomes are 40~160 nm in diameter and consist of phospholipid membrane and inner complex cargo (Figure 2). To identify exosomes, scientists have found some specific biomarkers on exosomes which can help to differentiate them from the other EVs. From then on, the cluster of differentiation (CD)9, CD63, and CD81 have been reported as membrane hallmarks of exosomes; notably, different exosomes may have a different cluster of biomarkers [68]. Besides the membrane protein, the exosomal content, such as the heat shock protein (HSP) family (e.g., HSP70, HSP90, and HSP72), which are enriched in cancer-derived exosome, can be used to identify the exosomes [10].

**Figure 3:**
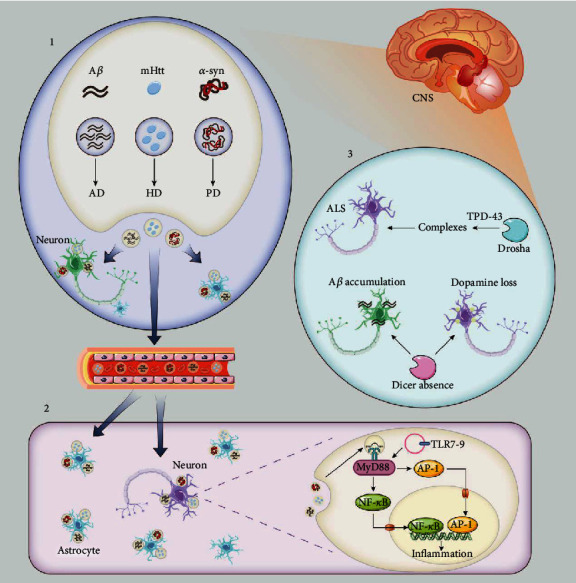
Exosomes and miRNA regulatory network in neurodegenerative diseases. (1) The major cause of AD, PD, and HD is abnormal aggregation of A*β*, *α*-syn, and mHtt in neurons. Neuronal cells can release exosomes into the extracellular space or transport them to surrounding cells via the bloodstream. (2) Exosomes contain miRNA. Following exosomes fuse with the membrane and release miRNA into the intracellular plasma membrane, TLRs are activated. TLR7-9 activates MyD88, which then activates NF-*κ*B and AP-1, leading to neuroinflammation and neuronal death. (3) The disorders in miRNA generation play role in neurodegenerative diseases, including absence of Dicer. The absence of Dicer contributes to A*β* accumulation and dopamine loss. TDP-43 could combine with Drosha, and it could be seen in ALS models. miRNAs: microRNAs; A*β*: amyloid-*β* peptide; *α*-syn: *α*-synuclein; mHtt: mutated Huntingtin; AD: Alzheimer's disease; PD: Parkinson's disease; HD: Huntington's disease; TLR: toll-like receptor; MyD88: myeloid differentiation factor NF-*κ*B, nuclear factor-*κ*B; AP-1: transcription factor activator protein-1; ALS: amyotrophic lateral sclerosis; TDP-43: transactivating response region DNA-binding protein 43.

Exosomes have a different pattern in generation from other EVs. Most EVs are generated by direct outward budding and fission of the plasma membrane. But the formation of exosomes is a three-step process. Firstly, plasma membrane forms the endocytic vesicles (endosomes); secondly, the inward budding of endosomal membranes results in some small vesicles, which, coupled with specific protein and nuclear acid, are then assembled into exosomes in a larger vesicle called multivesicular bodies (MVBs); and thirdly, the MVBs release the exosomes to the extracellular environment when fusing with the plasma membrane (Figure 2) [17, 67]. Once released, exosomes from different sources will be accepted by recipient cells and yield different effects depending on what they carry and what they target. Exosomes secreted by some kinds of cells such as MSCs will directly target the near cells and function as paracrine [69]. Some will be released to the blood and transferred to distant target cells with the blood circulation. Of particular interest here, the BBB prevents the CNS from diverse pathogenic factors while blocking the drug entry into the brain, making it hard to cure the CNS disease. Fortunately, exosomes have been demonstrated to have the potential to cross the BBB, enter the target cells, and realize intended functionality, indicating their therapeutic potential for CNS diseases (Figure 3) [67, 70, 71].

How exosomes are recognized and endocytosed by specific cells is an important aspect to be investigated. Although there is still no consensus concerning the main pathway by which EVs or a given EV subtype deliver content in the cytosol of specific acceptor cells, researchers have indeed found some evidence to support the specificity of exosome uptake. For example, exosomes released by cortical neurons upon synaptic activation bind exclusively to other neurons, not to glial cells [72]. Moreover, exosomes from oligodendrocytes tend to be selectively captured by microglia [73]. To date, there have been some assumptions explaining these specificities. The size and membrane components of exosomes may determine their recognition and engulfment by target cells [74, 75]. For instance, CD47 in exosome membranes can protect it from captured by macrophage and monocytes [76]. In addition, the membrane proteins of acceptor cells are also responsible for the recognition. Correspondingly, dendritic cell-surface CD11a and CD54 and exosome-surface CD9 and CD81 mainly mediate the targeting of exosomes to dendritic cells and the subsequent endocytosis [77]. Furthermore, although there is insufficient evidence that supports this view, the cargos that exosomes carry may also lead the exosome to target cell. For instance, a study by Nabet et al. showed that unshielded *RN7SL1* RNA in stromal-derived exosomes can stimulate a tumor-promoting pathway within a subset of breast cancer cells, which are primarily basal/triple-negative breast cancers [78]. The molecular mechanisms underlying exosomes' selectivity merit further research to uncover their bigger and more wide application prospects as option for targeted therapy.

### 3.2. Exosomal miRNAs

#### 3.2.1. Characteristics of Exosomal miRNAs

Recently, miRNAs have been identified in exosomes, which can be taken up by neighboring or distant cells and subsequently modulate recipient cells. miRNAs are a class of 17–24 nt small, noncodingRNAs, which mediate posttranscriptional gene silencing by binding to the 30-untranslated region (UTR) or open reading frame (ORF) region of target mRNAs [79]. There is growing evidence showed that exosomal miRNAs play an important role in disease progression, especially in neurodegenerative diseases [80–86]. miRNAs contribute to neurodegenerative diseases primarily by three pathways: (1) targeting the regulatory-related gene mRNA to inhibit the protein translation or degrade protein, (2) participating in neuroinflammation by directly binding to toll-like receptor or regulating its mRNA expression, and (3) yielding miRNA formation disorder [87]. Among these pathophysiologic processes, a tight interaction between miRNAs and oxidative stress has been revealed.

#### 3.2.2. Roles of Exosomal miRNAs in Neurodegenerative Diseases

*(1) Potential New Method for Gene Therapy*. Exosome is capable to transport functional miRNAs, and this endogenic carrier inspired people to replace virus-based gene therapy [88]. Compared with convention methods applied in RNA interference, exosomes have potential to be an ideal carrier of miRNAs for it can be up taken by recipient cell without evoking immune response and its ability to cross the blood-brain barrier [84]. Moreover, feasibility of exosome-based delivery system in miRNA treatment has been confirmed in animal model [89]. miRNA expression alters in different neurodegeneration disease, and some of them was proved to be involved in progression of diseases [90, 91]. Intervention regulating in these miRNAs with exosomes is also a new direction of gene therapy.

*(2) New Way of Intercellular Communication*. The most well-known intercellular communication mechanisms are chemical receptor-mediated event [92]. Exosome transportation between different cells broaden people's understanding on cell to cell communication. miRNAs, as one important cargo, also participate in intercellular communications [93]. For instance, exosomal miRNAs released from cancer cell could transfer functional information in paracrine level and influence the tumor microenvironment which includes various cells such as cancer-associated fibrosis and pericytes [94]. In addition, it has been reported that neurons can transport miRNAs by exosomes to astrocytes and in turn regulate protein expression of astrocytes indirectly [95]. In light of the central role played by astrocytes in the function of the CNS, it is not surprising that they have also been implicated in the onset and progression of neurodegenerative diseases. The above evidence suggested that neurons may regulate the protein expression of astrocytes through the secretion of exosomal miRNA, thereby involved in the pathophysiological process of neurodegenerative diseases. This may also provide a new direction for studying the relationship between exosomal miRNA and neurodegenerative diseases.

*(3) Biomarker for Diagnosis*. In neurodegenerative diseases, pathological changes occur several years before the onset of symptoms; thus, predictor for detection of diseases in early stage is always important but challenging. Concerning that exosomes have been recognized as nature carriers of miRNAs, its capability to cross the blood-brain barrier and miRNA expression altered in different neurodegenerative diseases; it is reasonable to infer exosomal miRNAs in peripheral blood change in different diseases. Exosomal miRNAs from blood sample of PD and AD patients were analyzed [96, 97]. Previous studies have already analyzed exosomal miRNA profiles in CSF of PD and AD patients, and its changes in miRNAs compared with healthy control were all observed [98, 99]. In the future, with miRNA profiles analyzed repeatedly and integration of different miRNA expresses data over time, exosomal miRNAs are likely to show its value as a biomarker of neurodegenerative diseases.

## 4. The Crosstalk between Exosomal miRNAs and Oxidative Stress in Neurodegenerative Diseases

As mentioned above, both oxidative stress and exosome-derived miRNAs are closely involved in neurodegenerative diseases. Intriguingly, oxidative stress can affect the expression levels of numerous miRNAs, and conversely, miRNAs are able to regulate manifold genes involved in the oxidative stress response as well [16]. Accordingly, oxidative stress and miRNA networks are inextricably intertwined during the neurodegenerative processes. In this section, we will introduce the crosstalk between exosomal miRNAs and oxidative stress in neurodegenerative diseases.

### 4.1. AD

miR-34a, a tumor suppressor transcript, is highly expressed in autopsied brain tissue of AD patients and has a strong pertinence to the pathogenesis of a cognitive disorder. During AD pathogenesis, miR-34a facilitates the amyloid precursor protein (APP) amyloidogenic processing, while miR-34a knockdown can inversely mitigate the APP accumulation in brain tissue [100, 101]. One mechanism would be the interaction between miR-34a and oxidative stress through the inhibition of the normal autophagy and the succeeding mitochondrial dysfunction, ultimately resulting in the aggregation of APP and progression of AD [102].

miR-141-3p, a potential serum plasma biomarker for Alzheimer's disease, has been reported to be observed with low concentrations in the plasma exosomes of Alzheimer's disease patients [97]. However, it has also been found to be abundant in exosomes of inflammation-stimulated astrocytes [103]. This phenomenon may explain the difference in pathological processes between acute and chronic neuroinflammation. miR-141-3p has also been shown to disrupt antioxidant defense systems, modulate mitochondrial function, and upregulate oxidative stress by inhibiting PTEN [104]. Nonetheless, its ability to affect oxidative stress was confirmed in human hepatocellular carcinoma cells. Therefore, it is necessary to verify its ability in neurons or glial cells.

miR-125b-5p is one of the most abundant microRNAs in the brain [105] and is predominantly expressed in neurons, astrocytes, and microglia [106]. Compared to healthy controls, upregulated miR-125b-5p was observed in cerebrospinal fluid-derived exosomes of patients with AD [107]. Evidences showed that over expression of miR-125b-5p can lead to significant hyperphosphorylation at T231/S235, which is related to progression of AD. [108] It was found that transfection with miR-125b significantly enhanced the apoptosis of neurons cells and phosphorylation of Tau by activation of cyclin-dependent kinase 5 (CDK5) and p35/25 [109]. Meanwhile, Lugli et al. [97] found that overexpression of miR-125b-5p can cause defective associative memory in mice. However, recent study found that miR-125b-5p can attenuate A*β*-induced oxidative stress. This effect is probably due to downregulation of the expression of beta-site amyloid precursor protein cleaving enzyme 1 (BACE1) [110]. Another study reported that inhibition of miR-125b-5p reduced ROS levels and lowered mitochondrial membrane potential, thereby demonstrating neuroprotective effects against oxidative stress [111]. The above evidence showed that miR-125b-5p may be a novel regulator of AD progress and could be as a therapeutic target for AD therapy.

### 4.2. PD

Studies in recent years have found that miR-34a appears to act in the neurotoxic pathways of PD-associated neurotoxins such as paraquat, rotenone, and 6-hydroxydopamine (6-OHDA). The mood stabilizing drug lithium chloride protects SH-SY5Y cells from paraquat-induced neurotoxicity by activating the antioxidant protein expression regulator nuclear factor 2-related factor 2 (NRF2) and miR-34a inhibition [112, 113]. Similarly, the dibenzocyclooctadiene lignin Schisandrin B, which is an antioxidant, reversed the 6-OHDA-induced increase in miR-34a expression and inhibition of NRF2 in cells [114]. Meanwhile, Ba et al. [114] also observed that behavioral improvement effected by Schisandrin B was reversed by lentiviral-mediated miR-34a overexpression in a 6-OHDA mouse PD model. In addition, stress conditions can induce increased miR-34a secretion in astrocytes [115].The release of miR-34a from astrocytes, delivered via exosomes, can enhance the sensitivity of dopaminergic neurons to neurotoxins by targeting Bcl-2 in a PD model [116]. Recent study also showed that upregulation of miR-34a can alleviate oxidative stress-induced neuronal apoptosis [117]. However, we still know little about what role does this effect plays in PD genesis for the moment. In view of the above findings and as far as cellular and animal models of PD are concerned, mounting evidence suggests that miR-34a has a pathophysiological role in PD.

MiR-137 is a highly conserved miRNA. It is enriched in Drosophila's brain and is reported upregulated in early PD flies [118]. Similar increase has been observed in the plasma of PD patients [119, 120]. Jiang et al. [121] revealed that downregulation of exosomal miR-137 can upregulate oxidation resistance 1 (OXR1), thereby exerting a neuroprotective effect against oxidative stress in PD mouse model.

Let-7 is a series of miRNAs which was first discovered in C. elegans and highly conserved across animal species. Disorders of Let-7 can lead to many diseases including neurodegenerative diseases, diabetes, and cancer [122]. Let-7 was reported to be overexpressed in PD model [123]. Previous report showed that upregulated exosomal Let-7 can be observed in the CSF of PD patients [98, 124], indicating that these miRNAs can be transported by exosomes. When exosomal Let-7 is taken up by neurons, it causes neurodegeneration through activation of toll-like receptor 7 (TLR7) [125]. Meanwhile, the Let-7 family has been reported to reduce the effects of leucine-rich repeat kinase 2 (LRRK2) functional mutations, which is involved in the pathogenesis of PD [126]. Moreover, in the C. elegans PD model, silencing of Let-7 leads to a mild increase in ROS levels, inducing neuronal autophagy, reducing the accumulation of *α*-synuclein protein, thereby alleviating disease progression in PD [127].

### 4.3. HD

Compared to AD and PD, relatively few studies have targeted the interaction between microRNAs and oxidative stress in ALS and HD. Downregulation of miR-124 expression is observed in both mouse models of HD and in the brains of human HD sufferers [128]. Cyclin A2 is one of the targets of miR-124, and Cyclin A2 expression increases as miR-124 expression decreases, which may reveal that miR-124 is involved in cell cycle dysregulation in HD cell models by regulating Cyclin A2 expression [129]. Recent studies have attempted to apply exosomal miR-124 as a therapy to alleviate HD symptoms in animal models. Although the result did not show an obvious improvement in HD animal symptoms, the feasibility of exosome-based miR-124 in an HD model was confirmed [89]. Though, there are a lack of studies specifically target on interaction between miRNA and oxidative stress in HD, given that oxidative stress can alter the expression levels of miRNAs [130], interaction between miR-124 and oxidative stress may play an important role in HD pathophysiological process and more research is needed in the future to confirm this.

### 4.4. ALS

Rizzuti et al. observed miR-34a expression downregulated in vitro model of ALS and confirmed the significant role of miR-34a in neurodegeneration and ALS [131]. Sirtuin 1 (SIRT1), one of the specific targets of miR-34a, is a protective factor against oxidative stress-induced apoptosis [132]. Along with downregulation of miR-34a, increased SIRT1 is also observed in vitro model of ALS [131]. This phenomenon suggests that inhibition of miR-34a can exert a protective role in ALS via increasing SIRT1 expression to against oxidative stress-induced apoptosis. In ALS, environmental signals can induce the liberation of free radicals, leading to oxidative stress and alteration of epigenetic mechanisms [133]. In addition, epigenetic modifications regulate miR-34a expression through demethylation of the promoter region of the miR-34a gene. Upregulation of miR-34a promotes the expression of TP53, which is associated with ALS, thereby activating multiple genes involved in the cell cycle [134].

miR-142-5p is a member of miR-142 family microRNAs. The decreased regulation of miR-142-5p in the CSF of ALS patients was reported [135]. Wang et al. found that inhibition of miR-142-5p can activate Nrf2, which in turn inhibits oxidative stress and cell damage via the OGD/R pathway [136]. Besides, this microRNA is also related to inflammation. Given the important role of inflammation and oxidative stress in ALS, validating the function of miR-142 in ALS will help expand our understanding of ALS pathogenesis and development.

## 5. Exosomal miRNAs as Biomarkers

Cell-derived active substances that can steadily be detected in cells, body fluids, or tissues are called molecular markers. Exosomal miRNAs in the body fluids of subjects can exist stably due to the fact that they are free from the degradation by ribonuclease (RNase), and further, they can be stably stored for 48 h at 4°C *in vitro* [137]. These characteristics favor the quality of the specimens before tested, underlying the clinical application of exosomal miRNAs as biomarkers of certain diseases. To date, many studies have attempted to utilize miRNAs as biomarkers for neurodegenerative disease (Table 1) [96–98, 107, 138–147].

### 5.1. Exosomal miRNAs as AD Biomarkers

Liu et al. identified a lower expression of exosomal miR-193b in the blood and cerebrospinal fluid (CSF) of AD patients compared with controls, indicating the potential of exosomal miR-193b as a unique and noninvasive biomarker for AD [138]. Moreover, significant differences in the expressions of miR-605-5p, miR-451a, miR-125b-5p, and miR-16-5p in the CSF-derived exosomes have been detected in young-onset AD patients [107]. Ting et al. assessed upregulated contents of miR-135a and miR-384 and downregulated miR-193b in the exosomes from AD patients' blood and suggested that serum exosomal miR-193b, together with miR-135a and miR384, could be utilized as reliable markers for AD [139]. Furthermore, another study revealed significant changes of 20 plasma exosome-derived miRNAs in the AD group; however, none of the aforementioned exosomal miRNAs was involved except miR-125b-5p [97]. These distinct findings may be due to the different techniques of separation and identification in each study [148], which implies further research, as well as unified standards, is warranted.

### 5.2. Exosomal miRNAs as PD Biomarkers

Gui et al. discovered that in the early stages of PD, there were 27 exosomal miRNAs derived from CSF of patients presenting with abnormal expression, among which miR-153, miR-409-3p, miR-10a-5p, and Let-7g-3p were significantly increased, while miR-1 and miR-19b-3p were significantly decreased, indicating their potential values as biomarkers for early diagnosis of PD [98]. In addition, downregulated miR-19b and upregulated miR-24 and miR195 in serum exosomes are also thought to serve as diagnostic markers for patients with PD [96]. Another study suggested that plasma exosomal miR-331-5p and miR-505 might represent promising biomarkers [145]. Similar to the AD-related research, these results are highly inconsistent. More control studies with larger samples are needed in the future to validate these miRNAs.

### 5.3. Exosomal miRNAs as HD Biomarkers

HD is a hereditary and slow-progressing neurodegenerative diseases. Diagnosis mainly relies on family genetic history and genetic testing. Although HD is an untreatable disease, biomarkers remain important to patients by providing early diagnostic clues or reflecting disease progression. Related researches found elevated levels of miR-100-5p and decreased levels of miR-330-3p and miR-641 correlate with total functional capacity in HD patients [149]. Gaughwin et al. [150] found significantly lower plasma miR-34b levels in presymptomatic HD patients compared to healthy controls, suggesting that miR-34b is a new potential biomarker for HD that can be stably expressed in plasma and detected before the onset of clinical symptoms. Moreover, it is reported that miR-124 expression is reduced in HD patients and can lead to upregulation of neuron-restrictive silencing factor (NRSF) expression, thereby suppressing the expression of brain-derived neurotrophic factors, suggesting that abnormal expression of miR-124 plays a key role in the pathogenesis of HD [129, 151, 152]. Although the use of exosome-based delivery method not significantly improving motor symptoms in an animal model of HD, it provides feasibility for exosomal miRNA-based treatment of HD [89].

### 5.4. Exosomal miRNAs as ALS Biomarkers

ALS is the most common and severe form of motor neuron disease in adults. The mechanism by which ALS occurs is currently unknown, and the lack of specific biomarkers makes clinical diagnosis difficult. Increasing evidence suggests that RNA metabolism including miRNAs may play an important role in the pathophysiological process of ALS. A global downregulation of miRNAs is a frequent molecular denominator for multiple forms of human ALS [153]. De Felice et al. [154] suggested that miR-338-3p was increased in peripheral leukocytes, serum, and cerebrospinal fluid (CSF) from sporadic ALS patients and considered the miRNA to be a potential biomarker for early diagnosis of sporadic ALS [149]. Meanwhile, plasma miR-130a-3p, miR-151b, and miR-221-3p levels were also decreased in patients with sporadic ALS and positively correlated with sporadic ALS progression, suggesting that these miRNA can be used not only as diagnostic biomarkers, but also for monitoring disease progression [155]. Moreover, in the later stages of ALS, increased expression levels of miR-155, miR-146a, and miR-124 further exacerbating the inflammatory response, leading to a disturbed intracellular environment and motor neuron degeneration and necrosis [156]. The above evidences demonstrate that exosomal miRNAs have potential biomarker functions of ALS.

## 6. The Promise of Exosome-Based Delivery of Therapeutic miRNA for Neurodegenerative Disease Therapy

The BBB has always been an impregnable obstacle to the therapeutic development of CNS disorders, hindering the clinical application of many promising agents [157]. As mentioned above, exosomes are able to cross the BBB [70]. Indeed, studies have found that exosomes have many additional advantages as a novel type of drug delivery vehicles. Specifically, they have low immunogenicity, high transport efficiency, and can inhibit inflammatory response as well as be administered over long distances [17, 157]. In addition, the small size of exosomes prevents them from phagocytosis of the mononuclear phagocyte system [158]. Although extensive preclinical models have been designed to investigate the value of the exosome-based delivery system for therapeutics, exosomes are still in the early stages of being used to treat neurodegenerative diseases [159]. Only one clinical trial using focused ultrasound delivery of intravenously infused exosomes to deliver growth factors and anti-inflammatory agents for the treatment of neurodegenerative dementias is being carried out (*ClinicalTrials.gov Identifier*: *NCT04202770*). Transcranial focused ultrasound administered immediately prior to exosome treatment is in an attempt to enhance the deployment of exosomes to the hippocampus of patients.

Exosome-based delivery of therapeutic miRNA for CNS diseases has become a hot research topic. Experimental studies have indicated that MSC-derived exosomes transferring functional miRNAs (e.g., miR-133b and miR-17-92 cluster) to neurons can promote neural plasticity and functional recovery after stroke [160–162]. Research has also been conducted to deliver exogenous miR-21 by MSC-derived exosomes to prevent nucleus pulposus cells from apoptosis and mitigate intervertebral disc degeneration [163]. Mechanically, miR-21 promotes cell survival possibly by binding to mRNA 3′ untranslated regions of *PTEN* to hinder its function, which activates Akt and Bcl-2 and suppresses Bad, Bax, and caspase-3, ultimately inhibiting cell apoptosis [164]. Similarly, exosomal-based delivery of exogenous functional miRNAs has the promise to be a novel therapeutic strategy for other CNS diseases, such as traumatic brain injury [165].

To date, a few preclinical studies have been carried out on the use of exosomal miRNA in the treatment of neurodegenerative disease. Lee et al. [89] transfected the miR-124 vector into HEK 293 cells to produce a cell line that stably expresses miR-124. These miR-124-overexpressed cells were cultured in Dulbecco's modified Eagle's medium without exosomes, and then the exosomes were harvested from these cells by an optimized protocol. Whereafter, exosomes encapsulated with miR-124 were injected into both striatums of HD models, and the results demonstrated that these exosomes exhibited high expression of miR-124 and successfully reduced the expression of target genes in recipient cells. Though this treatment did not significantly improve the behavioral symptoms of experimental animals, it has laid the foundation for the clinical application of exosome-based delivery of miRNA in neurodegenerative diseases. Considering the crosstalk between exosomal miRNAs and oxidative stress in neurodegenerative diseases, exosomal delivery of aforementioned miRNAs intertwined with oxidative stress may also hold therapeutic potential and merit further exploration.

Notably, although exosomes hold great promise as rational vehicles for RNA delivery, in particular miRNAs and/or siRNAs, the loading efficiency is limited. Recently, Li et al. [166] invented a novel strategy for loading therapeutic substances into exosomes. They fused exosomal membrane protein CD9 with RNA binding protein, which has a high affinity with miR-155, to enrich miR-155 into exosomes with a high loading efficiency. Hereby, encapsulated miR-155 could be effectively delivered to recipient cells and recognized endogenous targets. Moreover, enhanced loading efficiencies were also revealed by the exosomes enriched with the functional miRNA inhibitor and CRISPR/dCas9. These findings have shown the prospects these engineered exosomes hold for enhanced RNA cargo encapsulation.

In the future, more clinical studies verifying the clinical efficacy of exosome-based drug delivery systems as well as oxidative stress-associated exosomal miRNAs for neurodegenerative diseases and more preclinical studies exploring better methods for loading therapeutic miRNAs into exosomes are warranted.

## 7. Conclusion and Perspectives

In the past decades since exosomes and miRNAs were found in neurons, researchers have tried to explain cell-cell communication in CNS with an exosome cargo system. And through the cell-cell communication system, exosomes and miRNAs seem to have enormous potential in neurodegenerative diseases especially as we have found that exosomes have a cell specificity. Currently, the value of exosomes and exosome-derived miRNAs in neurodegenerative diseases have been extensively studied, such as early diagnosis of diseases through blood/CSF-based specific miRNA detection, and targeted therapy through exosomes carrying agents across the BBB. Notably, as an irreversible disease, preventive measures or ways to slow the progression of neurodegeneration tend to be more achievable. In this context, the oxidative stress response is one of the crucial targets. Some antioxidants have been used to treat neurodegenerative diseases and appear to have an impressive effect [4, 167]. Intriguingly, exosomal miRNAs can regulate manifold genes involved in oxidative stress, providing more ideas for revealing the oxidative stress response during neurodegenerative processes, yet there is not enough related research that uncovers the precise mechanisms. We can propose a reasonable assumption that miRNAs can build up a regulation network between neurons and other brain cells through an exosome cargo way and act on oxidative stress in different neurodegenerative diseases, which merits further research and may provide the basis for novel strategies of neurodegenerative disease management.

## Figures and Tables

**Table 1:** Exosomal miRNAs as candidate biomarkers for neurodegenerative diseases.

NDDs	Study groups^††^	Exosome source	RNA extraction	RNA identification	Exosomal miRNAs	Dysregulation	Efficiency of diagnosis	Ref.
AD	43 MCI; 51 DAT pts; number of NCs is not specified	Serum, plasma, and CSF	Total Exosome RNA Isolation kit	TaqMan qPCR method	miR-193b	DAT<MCI<NCs	/	[103]
	17 young-onset AD^†^; 12 NCs	CSF	miRCURY RNA Isolation Kit	RT-qPCR	miR-125b-5p	Upregulated	AUC = 0.723	[104]
					miR-16-5p, miR-451a, and miR-605-5p	Downregulated	AUC = 0.760, 0.951, and 0.706, respectively	
	13 late-onset AD^†^; 12 NCs	CSF	miRCURY RNA Isolation Kit	RT-qPCR	miR-125b-5p	Upregulated	AUC = 0.785	[104]
					miR-451a and miR-605-5p	Downregulated	AUC = 0.847 and 0.765, respectively	
	208 probable AD (101 MCI and 107 DAT) pts; 228 NCs	Serum	miRcute miRNA isolation kits	RT-qPCR	miR-135a and miR-384	Upregulated	3-miRNA signature: AUC = 0.997 (for MCI)	[105]
					miR-193b	Downregulated		
	35 pts; 35 NCs	Plasma	Differential centrifugation	Illumina deep sequencing	miR-185-5p, miR-342-3p, miR-141-3p, miR-342-5p, miR-23b-3p, miR-338-3p, and miR-3613-3p	Downregulated	83–89% accuracy	[106]
	19 pts; 44 NCs	CSF	Traditional TRIzol reagent	Small RNA sequencing	miR-27a-3p, miR-30a-5p, miR-34c-3p, piR_019949, and piR_020364	Upregulated	6-miRNA signature: AUC = 0.83	[107]
					piR_019324	Downregulated		
	10 pts; 10 NCs	CSF	miRCURY Exosome Isolation Kit	RT-qPCR	miR-9-5p and miR-598	Not significantly upregulated	/	[108]
	16 pts; 36 NCs	Serum	Plasma/Serum Exosomal RNA Isolation Kit	Small RNA deep sequencing and RT-qPCR validation	miR-361-5p, miR-30e-5p, miR-93-5p, miR-15a-5p, miR-143-3p, miR-335-5p, miR-106b-5p, miR-101-3p, miR-424-5p, miR-106a-5p, miR-18b-5p, miR-3065-5p, miR-20a-5p, and miR-582-5p	Upregulated	Sensitivity = 87%, specificity = 77% (adding established risk factors to the panel of deregulated miRNA^∗^)	[109]
					miR-1306-5p, miR-342-3p, and miR-15b-3p	Downregulated		
	5 AD pts; 5 vascular dementia controls	Serum	miRNeasy Mini Kit	TaqMan RT-PCR	miR-34b	Upregulated	/	[110]
	10 AD pts; 11 DLB controls; 11 NCs	Plasma	miRCURYTM RNA Isolation Kit-biofluids	Next generation sequencing and RT-qPCR	miR-451a and miR-21-5p	Downregulated, compared to DLB controls	AUC = 0.95 and 0.93, respectively	[111]^∗∗^
					miR-451a, miR-21-5p, miR-23a-3p, miR-126-3p, Let-7i-5p, and miR-151a-3p	Downregulated, compared to DLB controls	/	
					miR-183-5p, miR-24-3p, and miR-423-5p	Not significantly upregulated, compared to DLB controls and NCs	/	
PD	78 pts; NCs	CSF	Qiagen miRNeasy Serum/Plasma Kit	TaqMan Real-Time PCR	miR-153, miR-409-3p, miR-10a-5p, and Let-7g-3p	Upregulated	AUC: miR-153, 0.780; miR-409-3p, 0.970; miR-10a-5p, 0.900; combination of miR-153 and miR-409-3p, 0.990	[112]
					miR-1 and miR-19b-3p	Downregulated	AUC = 0.920 and 0.705, respectively	
	109 pts; 40 NCs	Serum	miRNeasy Mini Kit	RT-PCR followed by qPCR	miR-24 and miR-195	Upregulated	AUC = 0.908 and 0.697, respectively	[113]
					miR-19b	Downregulated	AUC = 0.753, sensitivity = 68.8%, specificity = 77.5%	
	52 pts; 48 NCs	Plasma	Exosomal RNA and Protein Extraction kit	RT-qPCR	miR-331-5p	Upregulated	AUC = 0.849	[114]
					miR-505	Downregulated	AUC = 0.898	
	5 PD pts; 5 vascular parkinsonism controls	Serum	miRNeasy Mini Kit	TaqMan RT-PCR	miR-29a	Upregulated	/	[110]
ASL	10 pts; 20 NCs	Serum	Traditional TRIzol reagent and Qiagen miRNeasy Mini Kit	RT-qPCR	miR-27a-3p	Downregulated	/	[115]
Dementia (AD and vascular dementia)	32 pts with dementia; 16 NCs	Serum	miRcute miRNA isolation kit	RT-qPCR	miR-223	Downregulated	AUC = 0.875	[116]

^∗^Risk factors included age, sex, and APOE *ε*4 allele status; deregulated miRNA panel was exclusive of miR-3065-5p. ^∗∗^MiRNAs in this study were derived from unspecified extracellular vesicles. ^†^Young-onset AD occurred before the age of 65 years; late-onset AD occurred after the age of 65 years. ^††^Only validation cohorts are listed in this table. Abbreviations: AD: Alzheimer's disease; ALS: amyotrophic lateral sclerosis; AUC: area under the curve; CSF: cerebrospinal fluid; DAT: dementia of Alzheimer type; DLB: dementia with Lewy bodies; MCI: mild cognitive impairment; miR/miRNA: microRNA; NCs: normal controls; NDDs: neurodegenerative diseases; PD: Parkinson's disease; piR: piwi-interacting RNA; pts: patients; RT-qPCR: quantitative reverse transcription polymerase chain reaction.

## Data Availability

N/A.

## Abbreviations

A*β*: Amyloid-beta
AD: Alzheimer's disease
ALS: Amyotrophic lateral sclerosis
APP: Amyloid precursor protein
BBB: Blood-brain barrier
CD: Cluster of differentiation
CNS: Central nervous system
COX: Cyclooxygenase
VSF: Cerebrospinal fluid
ETC: Electronic transfer chain
EVs: Extracellular vesicles
HD: Huntington's disease
HSP: Heat shock protein
mHtt: Mutant Huntingtin protein
miRNA: microRNA
MSC: Mesenchymal stem cell
mtDNA: Mitochondria DNA
MVBs: Multivesicular bodies
ncRNA: Noncoding RNA
PD: Parkinson's disease
PINK1: PTEN-induced putative kinase 1
ROS: Reactive oxygen species
SOD: Superoxide dismutase
TLR: Toll-like receptor.
